# Ethyl 2-[(2,4-difluoro­phen­yl)hydrazinyl­idene]-3-oxobutano­ate

**DOI:** 10.1107/S1600536812000803

**Published:** 2012-01-18

**Authors:** Hoong-Kun Fun, Ching Kheng Quah, Shobhitha Shetty, Balakrishna Kalluraya

**Affiliations:** a X-ray Crystallography Unit, School of Physics, Universiti Sains Malaysia, 11800 USM, Penang, Malaysia; b Department of Studies in Chemistry, Mangalore University, Mangalagangotri, Mangalore 574 199, India

## Abstract

The asymmetric unit of the title compound, C_12_H_12_F_2_N_2_O_3_, contains two mol­ecules, both of which exist in an *E* conformation with respect to their C=N bonds [1.321 (6) and 1.310 (6) Å]. The mol­ecular conformations are supported by intra­molecular N—H⋯O hydrogen bonds, which generate *S*(6) rings. In the crystal, mol­ecules are linked by C—H⋯O and C—H⋯F hydrogen bonds into layers lying parallel to (001). The crystal studied was an inversion twin with a 0.58 (1):0.42 (1) domain ratio.

## Related literature

For the biological activity of oxobutano­ate derivatives, see: Billington *et al.* (1979 ▶); Stancho *et al.* (2008 ▶). For the biological activity of pyrazole derivatives, see: Rai *et al.* (2008 ▶); Girisha *et al.* (2010 ▶); Isloor *et al.* (2009 ▶). For hydrogen-bond motifs, see: Bernstein *et al.* (1995 ▶). For bond-length data, see: Allen *et al.* (1987 ▶).
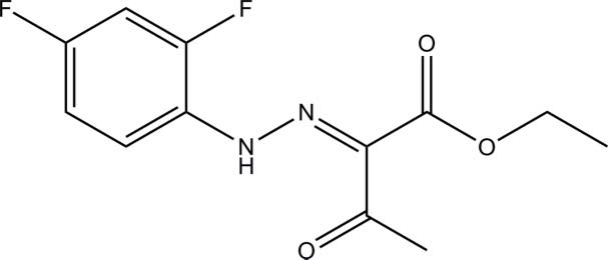


## Experimental

### 

#### Crystal data

C_12_H_12_F_2_N_2_O_3_
*M* *_r_* = 270.24
Orthorhombic, 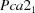

*a* = 21.814 (4) Å
*b* = 9.0079 (15) Å
*c* = 13.188 (2) Å
*V* = 2591.4 (8) Å^3^
*Z* = 8
Mo *K*α radiation
μ = 0.12 mm^−1^
*T* = 296 K
0.38 × 0.32 × 0.31 mm

#### Data collection

Bruker SMART APEXII DUO CCD diffractometer
Absorption correction: multi-scan (*SADABS*; Bruker, 2009 ▶) *T*_min_ = 0.957, *T*_max_ = 0.964
14829 measured reflections
3855 independent reflections
1903 reflections with *I* > 2σ(*I*)
*R*_int_ = 0.058

#### Refinement

*R*[*F* ^2^ > 2σ(*F* ^2^)] = 0.065
*wR*(*F* ^2^) = 0.219
*S* = 1.02
3855 reflections
356 parameters
1 restraint
H atoms treated by a mixture of independent and constrained refinement
Δρ_max_ = 0.22 e Å^−3^
Δρ_min_ = −0.24 e Å^−3^

### 

Data collection: *APEX2* (Bruker, 2009 ▶); cell refinement: *SAINT* (Bruker, 2009 ▶); data reduction: *SAINT*; program(s) used to solve structure: *SHELXTL* (Sheldrick, 2008 ▶); program(s) used to refine structure: *SHELXTL*; molecular graphics: *SHELXTL*; software used to prepare material for publication: *SHELXTL* and *PLATON* (Spek, 2009 ▶).

## Supplementary Material

Crystal structure: contains datablock(s) global, I. DOI: 10.1107/S1600536812000803/hb6590sup1.cif

Structure factors: contains datablock(s) I. DOI: 10.1107/S1600536812000803/hb6590Isup2.hkl

Supplementary material file. DOI: 10.1107/S1600536812000803/hb6590Isup3.cml

Additional supplementary materials:  crystallographic information; 3D view; checkCIF report

## Figures and Tables

**Table 1:** Hydrogen-bond geometry (Å, °)

*D*—H⋯*A*	*D*—H	H⋯*A*	*D*⋯*A*	*D*—H⋯*A*
N1*B*—H1*NB*⋯O2*B*	0.92 (4)	1.78 (4)	2.541 (5)	138 (4)
N1*A*—H1*NA*⋯O2*A*	0.86 (5)	1.88 (5)	2.547 (6)	133 (4)
C2*A*—H2*AA*⋯O2*A*^i^	0.93	2.58	3.476 (7)	163
C4*A*—H4*AA*⋯O3*A*^ii^	0.93	2.45	3.375 (6)	173
C2*B*—H2*BA*⋯O2*B*^i^	0.93	2.54	3.449 (6)	166
C4*B*—H4*BA*⋯O3*B*^ii^	0.93	2.46	3.380 (6)	170
C10*A*—H10*A*⋯F2*A*^iii^	0.96	2.54	3.343 (9)	141
C10*B*—H10*D*⋯F2*B*^iii^	0.96	2.48	3.330 (7)	148

Symmetry codes: (i) 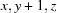; (ii) 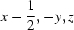; (iii) 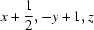.

**Fig. 1.**
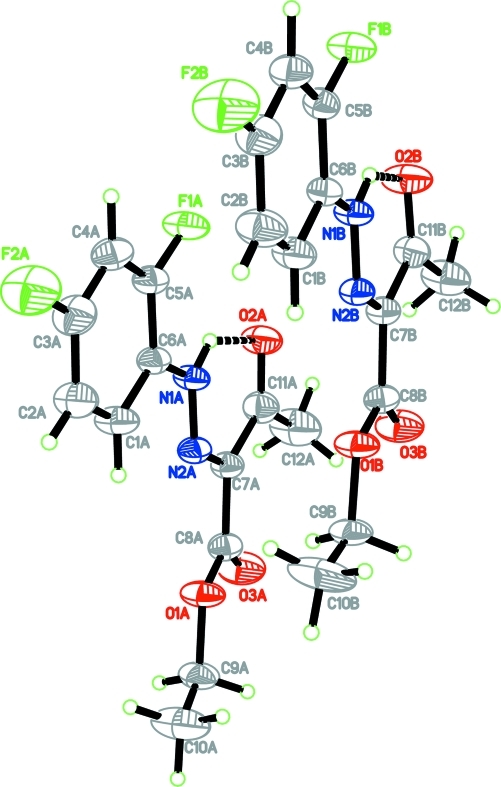
The molecular structure of the title compound showing 30% probability displacement ellipsoids for non-H atoms. Intramolecular hydrogen bonds are shown as dashed lines.

**Fig. 2.**
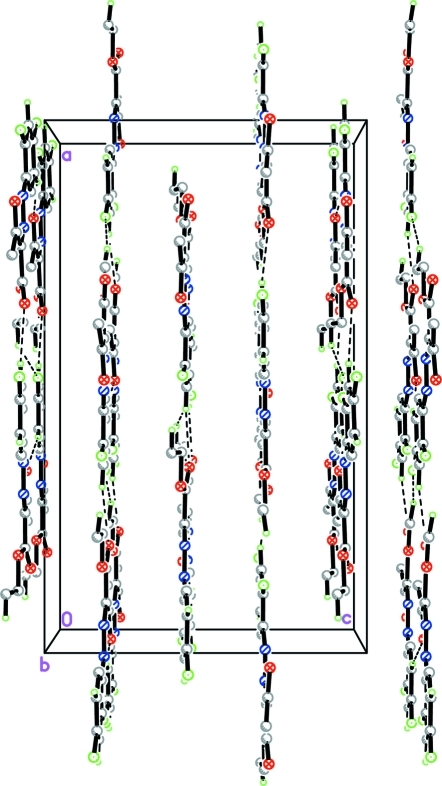
The crystal structure of the title compound, viewed along the b axis. H atoms not involved in hydrogen bonds (dashed lines) have been omitted for clarity.

## supplementary crystallographic information

### Comment

Derivatives of oxobutanoates are biologically important. 4-Methylthio-2- oxobutanoate was identified in the culture fluids of a range of bacteria, *e.g.* the yeast *Saccharomyces cerevisiae* and the fungus *Penicillium digitatum* (Billington *et al.*, 1979). Some oxobutanoates exhibit cytotoxic properties (Stancho *et al.*, 2008). Pyrazole derivatives are well established in the literatures as important biologically effective heterocyclic compounds (Rai *et al.*, 2008). These derivatives are the subject of many research studies due to their widespread pharmacological activities such as anti-inflammatory (Girisha *et al.*, 2010), antipyretic, antimicrobial (Isloor *et al.*, 2009), and antiviral activities. The widely prescribed anti-inflammatory pyrazole derivatives, celecoxib and deracoxib, are selective COX-2 inhibitors with reduced ulcerogenic side effects. The title compound (I), ethyl-2-[(2,4-difluorophenyl)hydrazinylidene]-3-oxobutanoate, is an intermediate in the preparation of pyrazole derivative. Condensation of oxobutanoate with thiosemicarbazide in glacial acetic acid medium gave the required pyrazole derivatives.

The asymmetric unit of (I) contains two independent molecules (Fig. 1), *A* and *B*, with comparable geometries. Both molecules exist in trans conformations with respect to the C7═N2 bonds [C7A═N2A = 1.321 (6) Å, C7B═N2B = 1.310 (6) Å]. The crystal studied was an inversion twin with a 0.58 (1):0.42 (1) domain ratio. The bond lengths (Allen *et al.*, 1987) and angles in the title compound are within normal ranges. The molecular structure is stabilized by intramolecular N1A–H1NA···O2A and N1B–H1NB···O2B hydrogen bonds which generate S(6) ring motifs (Bernstein *et al.*, 1995).

In the crystal (Fig. 2), molecules are linked *via* C2A–H2AA···O2A, C4A–H4AA···O3A, C10A–H10A···F2A, C2B–H2BA···O2B, C4B–H4BA···O3B and C10B–H10D···F2B hydrogen bonds (Table 1) into infinite sheets lying parallel to the (001) plane.

### Experimental

The title compound was prepared by dissolving 2,4-difluoroaniline (0.01 mol) in dilute hydrochloric acid (10 ml) and cooled to 273K in an ice bath. To this, a cold solution of sodium nitrite (0.02 mol) was added. The resulting diazonium salt solution was filtered into a cold solution of ethyl acetoacetate (0.05 mol) and sodium acetate in ethanol. The separated yellow solid was filtered, washed with water and recrystallized from ethanol. Yellow blocks of (I) were obtained from DMF by slow evaporation.

### Refinement

Atoms H1NA and H1NB were located in a difference Fourier map and refined freely with N-H = 0.86 (6) and 0.92 (5) Å. The remaining H atoms were positioned geometrically and refined using a riding model with C–H = 0.93-0.97 Å and *U*_iso_(H) = 1.2 or 1.5 *U*_eq_(C). A rotating-group model was applied for the methyl groups. The crystal studied was an inversion twin with a 0.58 (1):0.42 (1) domain ratio.

### Crystal data

**Table:**

C_12_H_12_F_2_N_2_O_3_	*F*(000) = 1120
*M**_r_* = 270.24	*D*_x_ = 1.385 Mg m^−^^3^
Orthorhombic, *P**c**a*2_1_	Mo *K*α radiation, λ = 0.71073 Å
Hall symbol: P 2c -2ac	Cell parameters from 3499 reflections
*a* = 21.814 (4) Å	θ = 2.3–29.8°
*b* = 9.0079 (15) Å	µ = 0.12 mm^−^^1^
*c* = 13.188 (2) Å	*T* = 296 K
*V* = 2591.4 (8) Å^3^	Block, yellow
*Z* = 8	0.38 × 0.32 × 0.31 mm

### Data collection

**Table:**

Bruker SMART APEXII DUO CCD diffractometer	3855 independent reflections
Radiation source: fine-focus sealed tube	1903 reflections with *I* > 2σ(*I*)
graphite	*R*_int_ = 0.058
φ and ω scans	θ_max_ = 29.9°, θ_min_ = 1.9°
Absorption correction: multi-scan (*SADABS*; Bruker, 2009)	*h* = −25→30
*T*_min_ = 0.957, *T*_max_ = 0.964	*k* = −12→11
14829 measured reflections	*l* = −18→18

### Refinement

**Table:**

Refinement on *F*^2^	Secondary atom site location: difference Fourier map
Least-squares matrix: full	Hydrogen site location: inferred from neighbouring sites
*R*[*F*^2^ > 2σ(*F*^2^)] = 0.065	H atoms treated by a mixture of independent and constrained refinement
*wR*(*F*^2^) = 0.219	*w* = 1/[σ^2^(*F*_o_^2^) + (0.1013*P*)^2^ + 0.7683*P*] where *P* = (*F*_o_^2^ + 2*F*_c_^2^)/3
*S* = 1.02	(Δ/σ)_max_ = 0.001
3855 reflections	Δρ_max_ = 0.22 e Å^−^^3^
356 parameters	Δρ_min_ = −0.24 e Å^−^^3^
1 restraint	Absolute structure: Flack (1983), 3406 Friedel pairs
Primary atom site location: structure-invariant direct methods	

### Special details

**Table:**

Geometry. All esds (except the esd in the dihedral angle between two l.s. planes) are estimated using the full covariance matrix. The cell esds are taken into account individually in the estimation of esds in distances, angles and torsion angles; correlations between esds in cell parameters are only used when they are defined by crystal symmetry. An approximate (isotropic) treatment of cell esds is used for estimating esds involving l.s. planes.
Refinement. Refinement of F^2^ against ALL reflections. The weighted R-factor wR and goodness of fit S are based on F^2^, conventional R-factors R are based on F, with F set to zero for negative F^2^. The threshold expression of F^2^ > 2sigma(F^2^) is used only for calculating R-factors(gt) etc. and is not relevant to the choice of reflections for refinement. R-factors based on F^2^ are statistically about twice as large as those based on F, and R- factors based on ALL data will be even larger.

### Fractional atomic coordinates and isotropic or equivalent isotropic displacement parameters (Å^2^)

**Table:**

	*x*	*y*	*z*	*U*_iso_*/*U*_eq_	
F1A	0.02111 (12)	−0.0552 (3)	0.4291 (4)	0.0738 (10)	
F2A	−0.01712 (16)	0.4553 (4)	0.4498 (5)	0.1088 (16)	
O1A	0.31436 (14)	0.0724 (4)	0.4235 (4)	0.0659 (10)	
O2A	0.16259 (16)	−0.2942 (4)	0.4261 (4)	0.0719 (11)	
O3A	0.34608 (17)	−0.1594 (5)	0.4477 (5)	0.0828 (14)	
N1A	0.14227 (16)	−0.0160 (5)	0.4336 (4)	0.0500 (10)	
N2A	0.20161 (16)	0.0028 (5)	0.4353 (4)	0.0478 (10)	
C1A	0.1253 (2)	0.2526 (6)	0.4428 (6)	0.0642 (16)	
H1AA	0.1673	0.2701	0.4442	0.077*	
C2A	0.0849 (3)	0.3703 (6)	0.4469 (6)	0.0700 (15)	
H2AA	0.0991	0.4675	0.4510	0.084*	
C3A	0.0232 (2)	0.3402 (6)	0.4448 (6)	0.0712 (16)	
C4A	−0.0002 (2)	0.2000 (6)	0.4385 (6)	0.0633 (13)	
H4AA	−0.0423	0.1829	0.4365	0.076*	
C5A	0.0411 (2)	0.0857 (6)	0.4353 (5)	0.0512 (11)	
C6A	0.10348 (19)	0.1081 (6)	0.4367 (4)	0.0469 (11)	
C7A	0.2391 (2)	−0.1124 (5)	0.4330 (5)	0.0483 (11)	
C8A	0.3050 (2)	−0.0713 (6)	0.4356 (5)	0.0529 (13)	
C9A	0.3782 (2)	0.1211 (7)	0.4260 (6)	0.0685 (16)	
H9AA	0.4024	0.0646	0.3779	0.082*	
H9AB	0.3952	0.1066	0.4932	0.082*	
C11A	0.2181 (2)	−0.2672 (6)	0.4285 (5)	0.0606 (13)	
C12A	0.2620 (3)	−0.3935 (6)	0.4238 (10)	0.104 (3)	
H12A	0.2397	−0.4853	0.4220	0.156*	
H12B	0.2880	−0.3917	0.4825	0.156*	
H12C	0.2866	−0.3849	0.3638	0.156*	
C10A	0.3790 (4)	0.2822 (9)	0.3988 (8)	0.113 (4)	
H10A	0.4204	0.3180	0.3999	0.169*	
H10B	0.3549	0.3369	0.4468	0.169*	
H10C	0.3622	0.2951	0.3321	0.169*	
F1B	−0.12910 (11)	−0.0552 (3)	0.1789 (3)	0.0686 (8)	
F2B	−0.16421 (16)	0.4584 (4)	0.1702 (5)	0.1085 (14)	
O1B	0.16419 (13)	0.0704 (4)	0.1812 (4)	0.0613 (10)	
O2B	0.01257 (17)	−0.2955 (4)	0.1951 (5)	0.0810 (14)	
O3B	0.19708 (15)	−0.1614 (4)	0.1938 (4)	0.0756 (12)	
N1B	−0.00755 (16)	−0.0181 (4)	0.1844 (4)	0.0491 (9)	
N2B	0.05230 (15)	−0.0004 (4)	0.1842 (4)	0.0479 (9)	
C1B	−0.0234 (2)	0.2521 (6)	0.1778 (6)	0.0622 (13)	
H1BA	0.0187	0.2693	0.1788	0.075*	
C2B	−0.0629 (2)	0.3694 (6)	0.1749 (7)	0.0731 (16)	
H2BA	−0.0482	0.4663	0.1725	0.088*	
C3B	−0.1250 (2)	0.3412 (6)	0.1756 (6)	0.0677 (15)	
C4B	−0.1493 (2)	0.2016 (6)	0.1751 (5)	0.0605 (13)	
H4BA	−0.1914	0.1856	0.1721	0.073*	
C5B	−0.1080 (2)	0.0857 (5)	0.1793 (5)	0.0489 (11)	
C6B	−0.04523 (19)	0.1069 (5)	0.1792 (5)	0.0445 (10)	
C7B	0.0894 (2)	−0.1147 (5)	0.1874 (4)	0.0465 (10)	
C8B	0.1558 (2)	−0.0748 (6)	0.1879 (5)	0.0486 (11)	
C9B	0.2268 (2)	0.1242 (6)	0.1836 (7)	0.0652 (14)	
H9BA	0.2501	0.0813	0.1281	0.078*	
H9BB	0.2463	0.0959	0.2469	0.078*	
C11B	0.0684 (2)	−0.2708 (6)	0.1888 (5)	0.0584 (12)	
C12B	0.1127 (3)	−0.3974 (6)	0.1820 (8)	0.0829 (18)	
H12D	0.0909	−0.4895	0.1883	0.124*	
H12E	0.1422	−0.3895	0.2357	0.124*	
H12F	0.1333	−0.3944	0.1178	0.124*	
C10B	0.2255 (3)	0.2861 (7)	0.1742 (12)	0.121 (3)	
H10D	0.2661	0.3249	0.1833	0.182*	
H10E	0.1988	0.3268	0.2249	0.182*	
H10F	0.2107	0.3128	0.1081	0.182*	
H1NB	−0.0208 (19)	−0.115 (5)	0.189 (4)	0.045 (12)*	
H1NA	0.127 (2)	−0.104 (6)	0.433 (5)	0.056 (15)*	

### Atomic displacement parameters (Å^2^)

**Table:**

	*U*^11^	*U*^22^	*U*^33^	*U*^12^	*U*^13^	*U*^23^
F1A	0.0420 (15)	0.066 (2)	0.114 (3)	−0.0146 (13)	−0.003 (2)	−0.001 (2)
F2A	0.070 (2)	0.079 (3)	0.177 (5)	0.0320 (18)	0.000 (3)	−0.003 (3)
O1A	0.0297 (15)	0.057 (2)	0.111 (3)	−0.0035 (15)	−0.002 (2)	0.005 (2)
O2A	0.052 (2)	0.059 (2)	0.104 (3)	−0.0139 (17)	0.005 (3)	−0.002 (3)
O3A	0.0406 (18)	0.073 (3)	0.134 (4)	0.0115 (18)	−0.006 (2)	0.008 (3)
N1A	0.0301 (16)	0.051 (2)	0.069 (3)	−0.0065 (17)	−0.004 (2)	−0.001 (2)
N2A	0.0309 (18)	0.056 (2)	0.056 (2)	−0.0023 (17)	−0.002 (2)	0.001 (2)
C1A	0.038 (2)	0.059 (4)	0.095 (4)	−0.007 (2)	0.000 (3)	0.005 (4)
C2A	0.055 (3)	0.058 (3)	0.098 (4)	−0.002 (2)	0.002 (4)	−0.002 (4)
C3A	0.053 (3)	0.066 (4)	0.095 (4)	0.019 (3)	0.002 (4)	−0.003 (4)
C4A	0.039 (2)	0.075 (4)	0.076 (3)	0.004 (2)	0.000 (3)	0.004 (4)
C5A	0.036 (2)	0.059 (3)	0.059 (3)	−0.004 (2)	−0.003 (3)	0.003 (3)
C6A	0.032 (2)	0.054 (3)	0.054 (3)	−0.0031 (19)	−0.002 (3)	0.001 (3)
C7A	0.036 (2)	0.048 (2)	0.061 (3)	−0.0008 (19)	0.000 (3)	0.000 (3)
C8A	0.037 (2)	0.061 (3)	0.061 (3)	−0.001 (2)	−0.002 (3)	0.003 (3)
C9A	0.033 (2)	0.076 (4)	0.097 (4)	−0.010 (2)	−0.003 (3)	−0.006 (4)
C11A	0.058 (3)	0.051 (3)	0.072 (3)	−0.002 (2)	0.000 (3)	0.003 (3)
C12A	0.077 (5)	0.049 (3)	0.185 (9)	0.007 (3)	0.004 (6)	−0.008 (6)
C10A	0.062 (4)	0.084 (5)	0.191 (11)	−0.021 (4)	0.000 (5)	0.006 (6)
F1B	0.0406 (15)	0.0605 (17)	0.105 (2)	−0.0105 (13)	−0.004 (2)	0.003 (2)
F2B	0.073 (2)	0.078 (2)	0.175 (4)	0.0353 (17)	0.001 (3)	0.003 (3)
O1B	0.0300 (15)	0.055 (2)	0.099 (3)	−0.0035 (13)	−0.001 (2)	0.003 (2)
O2B	0.048 (2)	0.057 (2)	0.139 (4)	−0.0121 (16)	0.006 (3)	0.004 (3)
O3B	0.0375 (17)	0.065 (2)	0.124 (4)	0.0095 (15)	−0.001 (2)	0.005 (3)
N1B	0.0292 (17)	0.046 (2)	0.072 (2)	−0.0036 (15)	−0.003 (2)	0.000 (3)
N2B	0.0308 (16)	0.058 (2)	0.055 (2)	−0.0046 (16)	−0.001 (2)	0.000 (2)
C1B	0.042 (3)	0.055 (3)	0.089 (4)	−0.005 (2)	−0.002 (3)	−0.007 (3)
C2B	0.051 (3)	0.056 (3)	0.112 (5)	0.002 (2)	−0.005 (4)	−0.001 (4)
C3B	0.052 (3)	0.059 (3)	0.093 (4)	0.015 (2)	−0.002 (4)	−0.007 (4)
C4B	0.041 (2)	0.069 (3)	0.072 (3)	0.002 (2)	−0.004 (3)	0.001 (3)
C5B	0.039 (2)	0.052 (3)	0.056 (3)	−0.001 (2)	−0.006 (3)	−0.001 (3)
C6B	0.033 (2)	0.050 (2)	0.051 (2)	−0.0036 (17)	−0.003 (3)	0.001 (3)
C7B	0.037 (2)	0.048 (2)	0.055 (3)	−0.0013 (19)	−0.001 (3)	0.003 (3)
C8B	0.036 (2)	0.052 (3)	0.058 (3)	0.0005 (19)	−0.001 (3)	0.006 (3)
C9B	0.028 (2)	0.075 (3)	0.093 (4)	−0.009 (2)	−0.001 (3)	0.000 (4)
C11B	0.051 (3)	0.055 (3)	0.069 (3)	−0.005 (2)	−0.001 (3)	−0.002 (3)
C12B	0.064 (3)	0.049 (3)	0.136 (5)	0.001 (3)	−0.009 (5)	0.002 (5)
C10B	0.069 (4)	0.063 (4)	0.232 (10)	−0.026 (3)	−0.017 (7)	0.000 (7)

### Geometric parameters (Å, °)

**Table:**

F1A—C5A	1.345 (6)	F1B—C5B	1.350 (5)
F2A—C3A	1.362 (6)	F2B—C3B	1.360 (6)
O1A—C8A	1.320 (7)	O1B—C8B	1.324 (6)
O1A—C9A	1.460 (6)	O1B—C9B	1.450 (5)
O2A—C11A	1.236 (6)	O2B—C11B	1.241 (6)
O3A—C8A	1.208 (6)	O3B—C8B	1.194 (6)
N1A—N2A	1.306 (5)	N1B—N2B	1.315 (5)
N1A—C6A	1.403 (6)	N1B—C6B	1.396 (6)
N1A—H1NA	0.86 (6)	N1B—H1NB	0.92 (5)
N2A—C7A	1.321 (6)	N2B—C7B	1.310 (6)
C1A—C2A	1.379 (8)	C1B—C2B	1.364 (8)
C1A—C6A	1.389 (7)	C1B—C6B	1.392 (7)
C1A—H1AA	0.9300	C1B—H1BA	0.9300
C2A—C3A	1.373 (8)	C2B—C3B	1.379 (7)
C2A—H2AA	0.9300	C2B—H2BA	0.9300
C3A—C4A	1.365 (8)	C3B—C4B	1.365 (8)
C4A—C5A	1.370 (7)	C4B—C5B	1.381 (7)
C4A—H4AA	0.9300	C4B—H4BA	0.9300
C5A—C6A	1.375 (6)	C5B—C6B	1.383 (6)
C7A—C11A	1.469 (7)	C7B—C11B	1.479 (7)
C7A—C8A	1.485 (7)	C7B—C8B	1.492 (6)
C9A—C10A	1.495 (10)	C9B—C10B	1.464 (8)
C9A—H9AA	0.9700	C9B—H9BA	0.9700
C9A—H9AB	0.9700	C9B—H9BB	0.9700
C11A—C12A	1.488 (8)	C11B—C12B	1.497 (7)
C12A—H12A	0.9600	C12B—H12D	0.9600
C12A—H12B	0.9600	C12B—H12E	0.9600
C12A—H12C	0.9600	C12B—H12F	0.9600
C10A—H10A	0.9600	C10B—H10D	0.9600
C10A—H10B	0.9600	C10B—H10E	0.9600
C10A—H10C	0.9600	C10B—H10F	0.9600
			
C8A—O1A—C9A	116.1 (4)	C8B—O1B—C9B	117.3 (3)
N2A—N1A—C6A	119.6 (4)	N2B—N1B—C6B	119.1 (4)
N2A—N1A—H1NA	120 (3)	N2B—N1B—H1NB	115 (3)
C6A—N1A—H1NA	121 (3)	C6B—N1B—H1NB	126 (3)
N1A—N2A—C7A	120.8 (4)	C7B—N2B—N1B	121.2 (4)
C2A—C1A—C6A	120.3 (5)	C2B—C1B—C6B	120.8 (5)
C2A—C1A—H1AA	119.9	C2B—C1B—H1BA	119.6
C6A—C1A—H1AA	119.9	C6B—C1B—H1BA	119.6
C3A—C2A—C1A	118.2 (5)	C1B—C2B—C3B	118.5 (5)
C3A—C2A—H2AA	120.9	C1B—C2B—H2BA	120.7
C1A—C2A—H2AA	120.9	C3B—C2B—H2BA	120.7
F2A—C3A—C4A	117.7 (5)	F2B—C3B—C4B	118.0 (5)
F2A—C3A—C2A	118.8 (5)	F2B—C3B—C2B	118.3 (5)
C4A—C3A—C2A	123.5 (5)	C4B—C3B—C2B	123.5 (5)
C3A—C4A—C5A	116.7 (5)	C3B—C4B—C5B	116.3 (4)
C3A—C4A—H4AA	121.6	C3B—C4B—H4BA	121.9
C5A—C4A—H4AA	121.6	C5B—C4B—H4BA	121.9
F1A—C5A—C4A	119.8 (4)	F1B—C5B—C4B	119.3 (4)
F1A—C5A—C6A	117.4 (4)	F1B—C5B—C6B	117.9 (4)
C4A—C5A—C6A	122.8 (5)	C4B—C5B—C6B	122.8 (4)
C5A—C6A—C1A	118.5 (5)	C5B—C6B—C1B	118.0 (4)
C5A—C6A—N1A	118.7 (4)	C5B—C6B—N1B	118.1 (4)
C1A—C6A—N1A	122.8 (4)	C1B—C6B—N1B	123.8 (4)
N2A—C7A—C11A	123.6 (4)	N2B—C7B—C11B	123.8 (4)
N2A—C7A—C8A	113.8 (4)	N2B—C7B—C8B	114.2 (4)
C11A—C7A—C8A	122.6 (4)	C11B—C7B—C8B	122.0 (4)
O3A—C8A—O1A	123.1 (5)	O3B—C8B—O1B	123.0 (4)
O3A—C8A—C7A	123.9 (5)	O3B—C8B—C7B	125.1 (5)
O1A—C8A—C7A	113.0 (4)	O1B—C8B—C7B	111.9 (4)
O1A—C9A—C10A	107.3 (5)	O1B—C9B—C10B	108.2 (4)
O1A—C9A—H9AA	110.3	O1B—C9B—H9BA	110.1
C10A—C9A—H9AA	110.3	C10B—C9B—H9BA	110.1
O1A—C9A—H9AB	110.3	O1B—C9B—H9BB	110.1
C10A—C9A—H9AB	110.3	C10B—C9B—H9BB	110.1
H9AA—C9A—H9AB	108.5	H9BA—C9B—H9BB	108.4
O2A—C11A—C7A	119.6 (5)	O2B—C11B—C7B	118.4 (5)
O2A—C11A—C12A	118.6 (5)	O2B—C11B—C12B	120.0 (5)
C7A—C11A—C12A	121.8 (5)	C7B—C11B—C12B	121.6 (4)
C11A—C12A—H12A	109.5	C11B—C12B—H12D	109.5
C11A—C12A—H12B	109.5	C11B—C12B—H12E	109.5
H12A—C12A—H12B	109.5	H12D—C12B—H12E	109.5
C11A—C12A—H12C	109.5	C11B—C12B—H12F	109.5
H12A—C12A—H12C	109.5	H12D—C12B—H12F	109.5
H12B—C12A—H12C	109.5	H12E—C12B—H12F	109.5
C9A—C10A—H10A	109.5	C9B—C10B—H10D	109.5
C9A—C10A—H10B	109.5	C9B—C10B—H10E	109.5
H10A—C10A—H10B	109.5	H10D—C10B—H10E	109.5
C9A—C10A—H10C	109.5	C9B—C10B—H10F	109.5
H10A—C10A—H10C	109.5	H10D—C10B—H10F	109.5
H10B—C10A—H10C	109.5	H10E—C10B—H10F	109.5
			
C6A—N1A—N2A—C7A	179.7 (6)	C6B—N1B—N2B—C7B	−178.9 (6)
C6A—C1A—C2A—C3A	0.0 (12)	C6B—C1B—C2B—C3B	1.4 (12)
C1A—C2A—C3A—F2A	179.3 (7)	C1B—C2B—C3B—F2B	−178.4 (7)
C1A—C2A—C3A—C4A	−0.2 (13)	C1B—C2B—C3B—C4B	−2.6 (13)
F2A—C3A—C4A—C5A	−178.9 (7)	F2B—C3B—C4B—C5B	179.2 (7)
C2A—C3A—C4A—C5A	0.7 (12)	C2B—C3B—C4B—C5B	3.3 (12)
C3A—C4A—C5A—F1A	−180.0 (7)	C3B—C4B—C5B—F1B	−179.9 (6)
C3A—C4A—C5A—C6A	−1.0 (10)	C3B—C4B—C5B—C6B	−3.0 (11)
F1A—C5A—C6A—C1A	179.8 (6)	F1B—C5B—C6B—C1B	178.9 (6)
C4A—C5A—C6A—C1A	0.9 (10)	C4B—C5B—C6B—C1B	1.9 (10)
F1A—C5A—C6A—N1A	−1.3 (9)	F1B—C5B—C6B—N1B	−3.5 (9)
C4A—C5A—C6A—N1A	179.7 (6)	C4B—C5B—C6B—N1B	179.5 (6)
C2A—C1A—C6A—C5A	−0.4 (10)	C2B—C1B—C6B—C5B	−1.1 (11)
C2A—C1A—C6A—N1A	−179.2 (7)	C2B—C1B—C6B—N1B	−178.5 (7)
N2A—N1A—C6A—C5A	−179.7 (5)	N2B—N1B—C6B—C5B	179.8 (6)
N2A—N1A—C6A—C1A	−0.8 (9)	N2B—N1B—C6B—C1B	−2.7 (10)
N1A—N2A—C7A—C11A	−0.1 (10)	N1B—N2B—C7B—C11B	1.7 (9)
N1A—N2A—C7A—C8A	−179.9 (5)	N1B—N2B—C7B—C8B	−179.3 (5)
C9A—O1A—C8A—O3A	−0.3 (10)	C9B—O1B—C8B—O3B	−1.4 (9)
C9A—O1A—C8A—C7A	179.6 (6)	C9B—O1B—C8B—C7B	178.4 (6)
N2A—C7A—C8A—O3A	169.7 (7)	N2B—C7B—C8B—O3B	177.5 (6)
C11A—C7A—C8A—O3A	−10.1 (11)	C11B—C7B—C8B—O3B	−3.4 (10)
N2A—C7A—C8A—O1A	−10.2 (9)	N2B—C7B—C8B—O1B	−2.3 (8)
C11A—C7A—C8A—O1A	170.0 (6)	C11B—C7B—C8B—O1B	176.8 (5)
C8A—O1A—C9A—C10A	173.4 (6)	C8B—O1B—C9B—C10B	179.4 (8)
N2A—C7A—C11A—O2A	0.5 (11)	N2B—C7B—C11B—O2B	−5.7 (10)
C8A—C7A—C11A—O2A	−179.8 (7)	C8B—C7B—C11B—O2B	175.2 (6)
N2A—C7A—C11A—C12A	178.7 (8)	N2B—C7B—C11B—C12B	173.8 (7)
C8A—C7A—C11A—C12A	−1.6 (12)	C8B—C7B—C11B—C12B	−5.2 (11)

### Hydrogen-bond geometry (Å, °)

**Table:**

*D*—H···*A*	*D*—H	H···*A*	*D*···*A*	*D*—H···*A*
N1B—H1NB···O2B	0.92 (4)	1.78 (4)	2.541 (5)	138 (4)
N1A—H1NA···O2A	0.86 (5)	1.88 (5)	2.547 (6)	133 (4)
C2A—H2AA···O2A^i^	0.93	2.58	3.476 (7)	163
C4A—H4AA···O3A^ii^	0.93	2.45	3.375 (6)	173
C2B—H2BA···O2B^i^	0.93	2.54	3.449 (6)	166
C4B—H4BA···O3B^ii^	0.93	2.46	3.380 (6)	170
C10A—H10A···F2A^iii^	0.96	2.54	3.343 (9)	141
C10B—H10D···F2B^iii^	0.96	2.48	3.330 (7)	148

Symmetry codes: (i) *x*, *y*+1, *z*; (ii) *x*−1/2, −*y*, *z*; (iii) *x*+1/2, −*y*+1, *z*.
